# Dark-field computed tomography reaches the human scale

**DOI:** 10.1073/pnas.2118799119

**Published:** 2022-02-07

**Authors:** Manuel Viermetz, Nikolai Gustschin, Clemens Schmid, Jakob Haeusele, Maximilian von Teuffenbach, Pascal Meyer, Frank Bergner, Tobias Lasser, Roland Proksa, Thomas Koehler, Franz Pfeiffer

**Affiliations:** ^a^Department of Physics, School of Natural Sciences, Technical University of Munich, 85748 Garching, Germany;; ^b^Munich Institute of Biomedical Engineering, Technical University of Munich, 85748 Garching, Germany;; ^c^Karlsruhe Institute of Technology, Institute of Microstructure Technology, 76344 Eggenstein-Leopoldshafen, Germany;; ^d^Medical Image Acquisition, Philips Research, 22335 Hamburg, Germany;; ^e^Computational Imaging and Inverse Problems, Department of Informatics, Technical University of Munich, 85748 Garching, Germany;; ^f^Institute for Advanced Study, Technical University of Munich, 85748 Garching, Germany;; ^g^Department of Diagnostic and Interventional Radiology, School of Medicine, Klinikum rechts der Isar, Technical University of Munich, 81675 Munich, Germany

**Keywords:** X-ray imaging, dark-field imaging, computed tomography, Talbot–Lau interferometry, X-ray small-angle scattering

## Abstract

X-ray computed tomography (CT) is one of the most commonly used three-dimensional medical imaging modalities today. It has been refined over several decades, with the most recent innovations including dual-energy and spectral photon-counting technologies. Nevertheless, it has been discovered that wave-optical contrast mechanisms—beyond the presently used X-ray attenuation—offer the potential of complementary information, particularly on otherwise unresolved tissue microstructure. One such approach is dark-field imaging, which has recently been introduced and already demonstrated significantly improved radiological benefit in small-animal models, especially for lung diseases. Until now, however, dark-field CT could not yet be translated to the human scale and has been restricted to benchtop and small-animal systems, with scan durations of several minutes or more. This is mainly because the adaption and upscaling to the mechanical complexity, speed, and size of a human CT scanner so far remained an unsolved challenge. Here, we now report the successful integration of a Talbot–Lau interferometer into a clinical CT gantry and present dark-field CT results of a human-sized anthropomorphic body phantom, reconstructed from a single rotation scan performed in 1 s. Moreover, we present our key hardware and software solutions to the previously unsolved roadblocks, which so far have kept dark-field CT from being translated from the optical bench into a rapidly rotating CT gantry, with all its associated challenges like vibrations, continuous rotation, and large field of view. This development enables clinical dark-field CT studies with human patients in the near future.

## Significance

X-ray computed tomography (CT) is one of the most commonly used diagnostic three-dimensional imaging modalities today. Conventionally, this noninvasive technique generates contrast by measuring the X-ray attenuation properties of different tissues. Considering the wave nature of X-rays, complementary contrast can be achieved by further measuring their small-angle scattering (dark-field) properties. This provides additional valuable diagnostic information on otherwise unresolved tissue microstructure. In our work, we have translated this wave-optical mechanism from the optical bench to a human-sized prototype CT system. This involved the integration of an interferometer into a clinical CT gantry and overcoming several associated challenges regarding vibrations, continuous gantry rotation, and large field of view. This development puts complementary X-ray contrast within reach for real-word medical applications.

Computed tomography (CT) provides high-resolution three-dimensional imaging with fast (subsecond) acquisition times for medical radiology (1). Its versatility allows for a wide variety of applications and thus, makes it an essential tool in diagnostic imaging. For example, just recently CT imaging proved to play a crucial role in clinical practice in the fight against COVID-19 (2). While very successful so far, present CT approaches [including the latest dual-energy and spectral approaches (3)] generate contrast solely based on attenuation differences in the tissue. As shown recently, significant additional diagnostic potential lies in also exploiting the complementary wave nature of X-rays, which results in refraction and small-angle scattering as complementary signal channels. A range of preclinical studies has proven that particularly the small-angle scattering (usually referred to as dark-field) signal is very valuable for the diagnosis and staging of lung diseases, such as chronic obstructive pulmonary disease (COPD) (4, 5), lung fibrosis (4, 6), pneumonia (7), and lung cancer (8). This is because the dark-field signal picks up complementary information on microstructural properties in lung parenchyma, which is beyond the resolution limit of presently used CT approaches (9, 10) and yet, does not require a higher dose than conventional CT.

X-ray dark-field CT, based on a grating interferometer, was originally introduced at highly brilliant synchrotron sources (11) and was then successfully translated to more readily available medical X-ray tube sources (12, 13). There have been a number of important technical advances in fabrication of the required grating optics (14–18), and new data processing approaches were introduced (19–23). More recently, first clinical prototypes have been developed for two-dimensional radiographic grating–based X-ray imaging, again supporting strongly the significant benefit of diagnostic information, particularly for lung diseases (24–27). The implementation into a human-scale CT system for first clinical studies, however, could so far not be achieved. This is essentially due to the markedly increased technological challenges associated with the required large field of view for human scanning, the cylindrical interferometer geometry, and the rapidly and continuously rotating gantry. Moreover, the fast rotation times (subseconds), with all the associated vibrational instabilities, posed many so far unresolved algorithmic challenges, which have not at all been addressed with presently existing benchtop or small-animal systems featuring step and shoot acquisition and typical acquisition times of several minutes to hours (4, 26).

Here, we now report on 1) a technological approach that enabled the construction of a dark-field CT system based on a compact, inverse, and cylindrically bent X-ray grating interferometer; 2) an associated algorithmic approach with a processing pipeline, which can compensate for inevitable vibrations in the fast-rotating CT system; and 3) results on a human-scale anthropomorphic thorax phantom acquired within a scan time of 1 s.

## System Design

There are several options to perform dark-field imaging. Commonly, the use of a Talbot–Lau interferometer is considered to be the most promising approach for dark-field imaging with respect to medical applications (28). The major challenge of implementing a Talbot–Lau interferometer into a CT gantry originates from the required X-ray optical gratings, which have typical features in the micrometer range and yet, must be arranged precisely over the full field of view. Other than the challenges regarding grating fabrication and mounting, their fine periods make the system extremely sensitive to drifts and vibrations, which are immanent as a CT gantry is a fast-rotating platform. While the gantry (i.e., the rotating part of the system) is decoupled from the surroundings by an air bearing (29), it still carries several vibrating components like pumps and the X-ray tube with a quickly rotating anode (30).

**Fig. 1.**
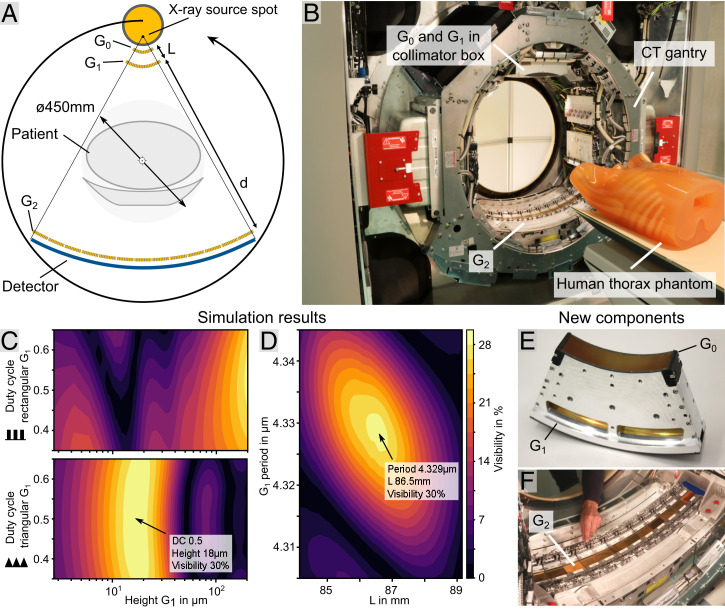
Design of the human-scale dark-field CT system. (*A*) Layout of the Talbot–Lau interferometer integrated into a conventional medical CT system. Bent gratings in an inverse geometry allow positioning of G_0_ and G_1_ close to the source. A large G_2_ is positioned close to the detector. Contrast formation is illustrated in *SI Appendix*, Fig. S1. (*B*) CT gantry equipped with a Talbot–Lau interferometer. The large G_2_ covering the detector is visible, while the G_0_ and G_1_ fixture is concealed by the collimator box. A human chest phantom is positioned on the patient couch. (*C*) Parameter analysis for rectangular and triangular G_1_ gratings. A maximum performance (i.e., fringe visibility) is expected for a duty cycle of 0.5 and 18.5 μm in height for a triangular profile. (*D*) Simulation shows that performance is highly parameter dependent. Small deviations in G_1_ periodicity in the nanometer range or in length *L* in the millimeter range cause irreversible performance loss. (*E*) A specialized G_0_ and G_1_ fixture to bend gratings to focus into the X-ray source spot. Rigid mounting is important to ensure stability during continuous rotation at high centrifugal forces. (*F*) Stitched G_2_ using a modular adjustment frame to individually position a total of 13 tiles. Fine position and rotation manipulation as well as long-time stability during rotation are key aspects of this component (*SI Appendix*, Fig. S2).

To maximize the sensitivity of the interferometer and simultaneously make the best use of the limited space on the gantry, we chose to position the three gratings in an inverse geometry (31), as shown in Fig. 1*A*. Gratings G_0_ and G_1_ are close to each other, and G_2_ is positioned on the opposite side of the bore. This allows for good use of the available space on the gantry and still retains the original CT bore diameter of 70 cm for the patient. The distances *L* and *d*, as well as the grating specifications, have been optimized to the setup-specific geometry and X-ray spectrum in Fresnel propagation-based simulations for maximum fringe visibility, which is a key performance parameter for dark-field imaging (15). Contrary to common Talbot–Lau interferometers, which use a rectangular G_1_ design, our simulation results in Fig. 1*C* show superior performance for a G_1_ grating with a triangular profile (32). Moreover, the simulations also reveal that, compared with common laboratory setups, the relatively large focal spot size of a medical high-performance X-ray source leads to significantly tighter tolerances for positioning and grating periods. As shown in Fig. 1*D*, deviations in the G_1_ period on the nanometer scale or deviations in the inter-grating distance *L* on the submillimeter scale will cause a significant visibility loss. The presented simulations use a source size of 1.1 mm. For high stability and precise positioning, special grating mounts shown in Fig. 1 *E* and *F* have been developed. The curvature of these mounts focuses into the X-ray source spot, which adapts the interferometer to the extremely divergent beam geometry (33). This requires bending of the gratings as they are manufactured on a flat substrate.

The gratings G_0_ and G_1_, which are mounted together, are implemented into the collimator box of the CT system. The grating G_2_ is installed directly in front of the detector as shown in Fig. 1 *B* and *F* and *SI Appendix*, Fig. S2. The largest component of the interferometer is the G_2_ grating, which must cover about 80 cm of arc length in order to support a field of view of 45 cm. Gratings of such size can currently only be combined from several smaller tiles, as shown in Fig. 1*F* and *SI Appendix*, Fig. S2. This increases the complexity of the system because all individual gratings must be carefully aligned to each other, but this allows replacement of tiles if required.

## Optimization and Characterization Procedure

**Fig. 2.**
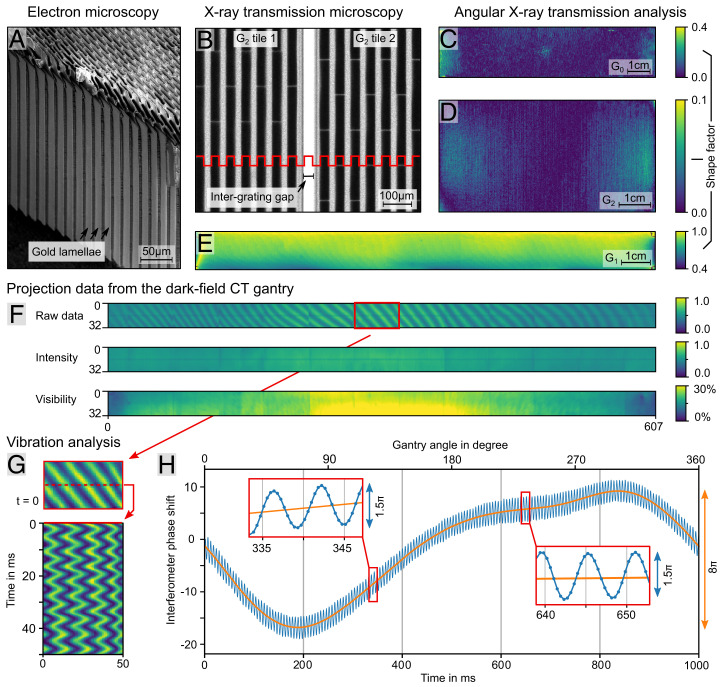
Key performance parameters and vibrational analysis. (*A*) Scanning electron microscopy image of a G_0_ grating fragment. Residual resist structures including support bridges are visible at the top. With a mean height of 280 μm and a period of 4.8  μm, it exhibits a high aspect ratio of ∼115. In *SI Appendix*, Fig. S3, a rendering is shown to visualize the high aspect ratio. (*B*) High-resolution X-ray microscopy of a G_2_ stitching gap between two tiles. (*C*–*E*) Quality evaluation of the G_0_, G_1_, and an example G_2_ tile using AXT analysis. The shape factor is a measure directly correlated to the quality of the grating. The factor is zero for perfectly rectangular gratings (G_0_ and G_2_) and one for perfectly triangular gratings like G_1_. The G_2_ tiles have consistent quality and only minor defects. G_0_ and G_1_ are mostly of fine quality, with defects toward the left and right edges. These defects are tolerable as the layout width is slightly larger than necessary. (*F*) Sample free projection data show the typical Moiré fringes in the raw data. By processing of the raw data, intensity and visibility images are generated. Visibility directly correlates to the performance of the interferometer, and the measured peak visibility of up to 30% is a good result considering the hard X-ray spectrum of 80 kVp. A plot of the column mean visibility is shown in *SI Appendix*, Fig. S4. (*G*) Analysis of the raw data sinogram showing oscillation of the fringes over time. A full sinogram can be found in *SI Appendix*, Fig. S5. (*H*) Detailed analysis of the oscillation shows angular dependence and a high-frequency vibration. The angular dependence (orange curve) is reproducible from scan to scan. The *Insets* illustrate that the high frequency is well sampled by the fast exposure time as the peak-to-peak amplitude is limited to 1.5π.

Due to the typical energies used in clinical CT systems (80 to 120 kVp), the fabrication of the fine grating structures for a Talbot–Lau interferometer is extremely difficult, as very high absorbing structures (>200  μm) yet with feature sizes of a few micrometers are required. Even though gold is used as the absorber material because of its high X-ray absorption cross-section, the fine periodic lines must still exhibit a height of more than 200 μm for reasonable performance. For fabrication of such high–aspect ratio structures, we chose the deep X-ray LIGA (German for “Lithographie, Galvanik, Abformung”) process (14, 34), as it outperforms silicon etching–based methods (aspect ratios < 80) (18, 35) and is applicable to grating substrates suitable for bending (16, 17, 33). In Fig. 2*A*, an example of a 280-µ m-high grating structure with an aspect ratio exceeding 100 demonstrates the most recent LIGA fabrication results. In order to closely monitor and improve the fabrication process at this level and to ensure coverage of the whole detector surface with sufficient grating quality and low defect rate, we have developed a dedicated angular X-ray transmission (AXT) analysis, which extends the standard quality control process (36). For that purpose, we assign a quality shape factor to characterize the fabricated gratings. As depicted in Fig. 2 *C*–*E*, the gratings mostly are of excellent quality, with only some defects limited to the outer left and right areas of G_0_ and G_1_. Since these areas do not contribute to the required field of view, they do not affect the overall system performance.

Recent advances in the fabrication process now allow for manufacturing G_0_ and G_1_ in the required dimensions and small bending radii, which reduce potential errors during assembly and increase the stability. However, the much larger G_2_ must still be combined from multiple separate tiles, which is realized by a specialized modular mount. It allows us to combine and adjust the bent grating tiles inside the gantry with a gap size comfortably below 100 μm and to maintain a continuous phase across the tiles at the same time to avoid stitching artifacts (17). An example is presented in a high-resolution transmission image of a stitching gap in Fig. 2*B*, where the red line indicates the continuous period.

To assess the system performance and stability, a test dataset without a sample is analyzed here. In Fig. 2*F*, the raw data output from the detector shows the expected Moiré fringe pattern. As a measure of the setup performance, we analyze the intensity *I*_0_ and visibility (13) *V*_0_, as shown in Fig. 2*F*, which are retrieved by processing the raw projection data. The intensity variation across the detector is a desired effect, which optimizes the applied radiation dose. While usually achieved with a bowtie filter in conventional clinical CT, here it is caused by partial shadowing of the X-ray beam in G_0_, which increases toward larger fan angles from the center of the detector. Due to the large source spot size and the small acceptance angle of the high–aspect ratio G_0_, an increasing fraction of the radiation traverses the grating structure nonperpendicularly and thus, is attenuated. Additionally, this leads to a degeneration of the slit sources for larger fan angles (i.e., where the X-ray source spot is larger) (30). This effect leads to a visibility decrease toward the left and right as can be seen in the visibility image in Fig. 2*F*. Nevertheless, the most important central region performs well with a maximum visibility of 26% in the center, which is close to the simulation results shown in Fig. 1. Including the outer regions, where the visibility drop due to the partial shadowing in G_0_ reduces the performance (a plotted version is in *SI Appendix*, Fig. S4), the mean visibility of the interferometer is around 20%.

To analyze the setup stability, a sinogram in Fig. 2*G* demonstrates how the fringe pattern oscillates during the measurement. Note that the fringe phase changes by 2π if any of the gratings are moved relative to the others by its full period. Mechanical parts, such as fans, the cooling system, and the anode drive, cause vibrations of the interferometer, which induce the observed oscillation of the fringes. While this frequency is defined by the exciting mechanical components, the resulting amplitude is configurable by the stiffness of the grating mounts. Fig. 2*H* shows an analysis of the oscillation over one full rotation. In addition to the high-frequency oscillation, a slow phase change coupled to the angular position of the gantry is observed. This low-frequency phase change is identical in every rotation and originates presumably from deformations of the system caused by gantry imbalance as well as the centrifugal and gravitational forces.

## Algorithmic Approach

In contrast to the typically employed step and shoot approaches (11) and the slow rotation times in benchtop laboratory setups or small-animal systems, our rapidly rotating clinical CT system causes a large variety of drifts and vibrations, leading to strong intensity, visibility, and phase fluctuations as shown in Fig. 2. Therefore, all so far developed processing methods cannot be applied since they cannot, for example, deal correctly with local intensity variations, which are among the major cause of artifacts we found during development of this dark-field CT system.

**Fig. 3.**
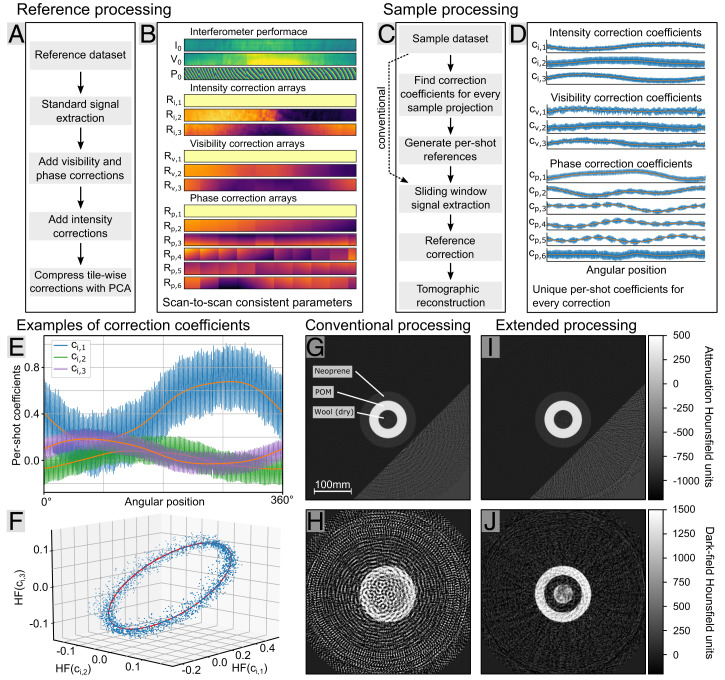
Data processing strategy. (*A*) Reference processing pipeline to extract scan-to-scan persistent system characteristics from an air scan. Here, we introduce local intensity fluctuation corrections and information compression using PCA. (*B*) Interferometer performance and correction array results from reference processing. (*C*) The sample processing pipeline based on sliding window processing. As the high-frequency oscillations differ from the reference scan, we use an optimization step to identify the optimal linear combination of the correction arrays to estimate the current sample free fringe parameters and thus, to suppress vibration artifacts. (*D*) Results of the correction coefficient optimization using prior knowledge from the reference scan. (*E*) Coefficients of the three intensity correction arrays. The angular position-dependent drift (orange) is scan-to-scan consistent and can be utilized as prior knowledge in the correction optimization step. (*F*) Three-dimensional scatterplot of the three intensity correction coefficients after subtraction of the low-frequency component shown in *E*. A correlation can be observed, which can also be used during the optimization step as prior knowledge. (*G* and *H*) Attenuation and dark-field FBP reconstructions after conventional sliding window processing, respectively. Both modalities suffer from vibration artifacts, as the high frequency is not corrected during processing. (*I* and *J*) Attenuation and dark-field FBP reconstruction after extended processing, respectively, which successfully removes vibration artifacts, giving a clearer reconstruction in both modalities. For better visualization of the background, the lower right corners in *G* and *I* are shown in a modified color range between –1,200 and –600 Hounsfield units.

We, therefore, developed an algorithmic approach for the data processing, which can cope with the various challenges laid out above. As illustrated in Fig. 3 *A* and *C*, we analyze a sample free reference scan to extract scan-to-scan persistent system characteristics, such as the mean performance, and use linear combinations of correction arrays to model the fluctuations in intensity, visibility, and phase in each individual detector reading. Fig. 3*A* lists the different reference processing steps, and Fig. 3*B* shows the resulting system characteristics. The only information that is not scan-to-scan persistent is the high-frequency component (around 175 Hz) of the correction array coefficients, which are plotted in Fig. 3*E* as an example for the three intensity correction arrays. Only the low-frequency component, plotted in orange, is persistent among different scans, and therefore, further extensions are necessary during the sample processing.

Ignoring the high-frequency component of the coefficients leads to extreme artifacts in the reconstructed images as shown in Fig. 3 *G* and *H*. To solve this problem, we developed an iterative optimization to retrieve the high-frequency component, which is ambiguous because sample information and high-frequency oscillation can coincide. To suppress this cross-talk effect, we regularize to prior knowledge like the expected frequency and amplitude of the variation as well as other characteristics, which are scan-to-scan persistent, like correlations between the different coefficients. In Fig. 3*F*, the high-frequency components of the three intensity correction coefficients are plotted, revealing their correlation as they are constrained onto an elliptical curve.

After this optimization, an individual reference correction for every sample projection is generated. It includes both the low- and high-frequency components of the correction coefficients, which are shown by the blue plots in Fig. 3*D*. The subsequent tomographic reconstruction with filtered back projection (FBP) (37) shown in Fig. 3 *I* and *J* is now free of vibration-induced artifacts. The remaining low-frequency noise-like structures in the dark-field image are due to motion artifacts. With more advanced processing methods, this artifact can be effectively suppressed (22).

All scans presented in this paper are single-rotation axial scans measured at 1 s rotation time. Since the gantry rotates continuously, a sliding window approach (19) is applied for the signal extraction. While vibrations in an interferometer generally only cause difficulties, our setup actually leverages the induced high-frequency phase variation. As demonstrated in Fig. 2*H*, the vibration guarantees relatively adequate phase sampling over at least 1.5π within comparably small window sizes of only 10 projections. If this intrinsic phase oscillation was absent, we would require an actuator to displace one grating during the scan, similar to a regular phase-stepping approach (11). The total duration from data acquisition to finished sample reconstruction is below 15 min.

## First Results

**Fig. 4.**
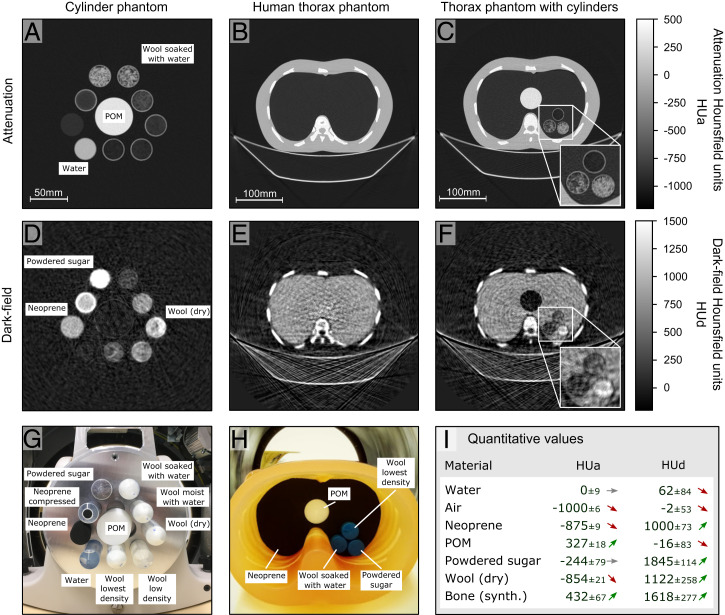
Human-scale dark-field CT results. (*A*–*C*) Conventional (attenuation) tomograms of a cylinder phantom and a modified human chest phantom with two different inserts. They are free of artifacts and allow us to easily distinguish different absorbing materials, like bones, soft tissue, and air. (*D*–*F*) Respective dark-field reconstructions showing the scattering power of the different materials. The dark-field signal clearly is of complementary nature, and we even observe contrast reversal for materials with high-scattering but low-density values (e.g., neoprene or cotton wool). The zoomed *Insets* in *C* and *F* highlight the area with additional material inserts. (*G* and *H*) Photographs of the cylinder phantom and the clinical human chest phantom with a black neoprene insert, respectively. (*I*) Table of different materials HUa and HUd from the reconstruction shown in *A* and *D*, including their SDs. The arrows indicate a qualitative classification of the signal within the overall measured signal range of the respective contrast modality. Quantitative multimodal imaging allows for an extended perception of CT images and will be beneficial for various diagnostic tasks. The enhanced material differentiation based on attenuation and scattering is particularly promising for porous and low-density materials (e.g., neoprene, powder materials, or fibrous materials like wool) and thus, has the potential to provide significant benefit for lung diagnostics.

To assess the quantitative performance of this first human-scale prototype dark-field CT system, we used a phantom composed of different materials in plastic tubes (Fig. 4*G*). The reconstructed attenuation and dark-field images are shown in Fig. 4 *A* and *D*, respectively. It is possible to differentiate not only the strongly attenuating water and polyoxymethylene (POM) from the less dense (i.e., less attenuating) materials but also, the small-angle scattering of X-rays induced by these materials. The dark-field image channel allows us to differentiate foams, powders, and cotton wool compositions since the dark-field signal picks up information on fine structures and porosities (9, 38).

To evaluate the system regarding potential future clinical application, we employed a modified human chest phantom based on a commercially available product (Lungman; Kyoto Kagaku), which represents a chest torso with a girth of 94 cm. To simulate lung tissue, we inserted pieces of neoprene, whose porosity models the microstructure of the lung alveoli and is a suitable substitute (*SI Appendix*, Fig. S6) (38). We also prepared plastic tube inserts, as shown in Fig. 4*H*, to evaluate additional materials. Fig. 4 *B* and *C* displays the conventional attenuation reconstructions with the simple neoprene and multimaterial inserts. The sharp and artifact free attenuation images show the expected good contrast for bones along with a very weak signal from the lung area, which is a typical feature of lung tissue. In the corresponding dark-field CT images (Fig. 4 *E* and *F*), we can observe that complementary information on the scattering properties of the foam can be visualized. The porous neoprene and fine-structured sugar stand out in the dark-field channel, while homogenous materials, like POM and soft tissue, generate no signal. The synthetic bones of the phantom also appear to have a notably strong small-angle scattering characteristic.

Particularly from the magnified regions of Fig. 4 *C* and *F*, it is evident that the dark-field modality features a lower resolution compared with the attenuation image. This is caused by a blurring effect inherent to the sliding window signal extraction approach and could be eliminated by model-based iterative reconstruction methods (22). However, it is important to point out that the dark-field signal shows subresolution bulk tissue properties, independent of the actual spatial resolution of the tomogram, which relativizes the need for high-resolution dark-field CT.

Based on these results, we conclude that our dark-field CT prototype can retrieve valuable subresolution structure information in a clinical setting. The combination of attenuation and dark-field contrast can facilitate a quantitative differentiation of specific tissue types, which cannot be achieved by attenuation only. In analogy to the Hounsfield units, which are used as a quantitative measure for attenuation CT (we refer to them as HUa), we propose the following equation to transform the measured linear diffusion coefficient *ε* (39–42) to the dark-field Hounsfield unit (HUd) scale for quantitative dark-field CT imaging (4):

$$\text{HUd}\;\left(x\right)=1,000\cdot \frac{\varepsilon (x)-\varepsilon_{0}}{\varepsilon_{\text{Neoprene}}-\varepsilon_{0}}.$$

This scale is calibrated to $\varepsilon_{\text{Neoprene}}$ as a dark-field reference material, and $\varepsilon_{0}$ is equal to zero for air as a nonscattering reference. The quantitative dark-field imaging results from our prototype scanner are shown in Fig. 4*I* (*SI Appendix*, Fig. S7 shows an extended table). The arrows qualitatively indicate the strength of the signal in the respective contrast modality and demonstrate that the different combinations of both quantitative scales, HUa and HUd, enable a clearer identification of physical sample properties.

## Discussion

In summary, we demonstrated a dark-field CT system, which can image the size of a human thorax with a field of view of 45 cm in a single second. All so far remaining technical challenges have been overcome, and solutions have been combined into one system. Namely, the large field of view is achieved by bent grating structures for high X-ray energies, which are rigidly mounted on a clinical CT gantry, and a processing approach, which compensates for vibration-induced artifacts.

This work is focused on the demonstration that dark-field CT based on a standard medical CT platform is technically feasible. Our results are hence limited to phantom studies and small coverage. Currently, we proceed with increasing the reconstruction volume of a single scan by extending to helical acquisition and reconstruction. Helical acquisition is required to obtain the coverage of the entire lung area during one breath hold. This is needed to meet clinical requirements regarding usability, acquisition time, and radiation dose for regulatory approval of the first in vivo patient studies.

For the innovations in clinical CT, this presented implementation of a Talbot–Lau interferometer on a continuously rotating gantry is a major milestone. Particularly, the fact that the demonstrated proof of concept requires only minor modifications of a prevalent device allows for rapid translation to clinical application and commercialization. We expect that similar dark-field implementations are feasible in the majority of existing CT gantries. Furthermore, the proposed dark-field CT is not only fully compatible but actually, even advantageous in combination with other innovations (e.g., dual-energy and photon-counting detectors) that are currently introduced by the manufacturers (43). Consequently, we see grating-based dark-field imaging on the road map for future CT innovation.

For clinical realization, we propose the HUd scale for the dark-field signal. By having the conventional HUa and the additional HUd reconstructions side by side, we are confident that the further diagnostic information can be interpreted efficiently. In MRI, a similar duality of contrast mechanisms, namely T1 and T2, is already well known and successfully applied in clinical routine. After dark-field CT systems are approved and available in clinics, we expect an immediate impact in lung imaging (4, 27), as this has already been demonstrated in previous small-animal studies on COPD, emphysema, fibrosis, and lung cancer. Potential further clinical applications include foreign body detection (44) and characterization of trabecular bone microstructure (45) and calcifications as they can occur in tissue or as plaques in blood vessels, as well as differentiation between different types of kidney stones (46). In the context of clinical translation, the presented work represents the final milestone before the first patient study.

## Materials and Methods

### The Principle of Talbot–Lau Interferometry

An X-ray grating interferometer is used to measure X-ray attenuation, refraction, and small-angle scattering simultaneously (12, 13). The gratings are patterns composed of many parallel absorbing lines at a fixed periodicity (14, 16, 17). Grating G_1_ introduces a fine intensity modulation on the incident radiation with a periodicity in the range of a few micrometers. To resolve this line pattern, a highly absorbing analyzer grating G_2_ is positioned in front of the detector. Hence, the subpixel-sized intensity modulation is converted by the Moiré effect to a low-frequency fringe pattern resolvable by the detector. An additional source grating G_0_ splits the radiation from an incoherent X-ray source into many slit sources, which fulfill the coherence requirement for this method (12). Attenuation of the radiation by the sample causes a decrease of intensity, while refraction and small-angle scattering induce small distortions and contrast change of the generated fringe pattern, respectively. *SI Appendix*, Fig. S1 shows a simplified illustration of the interaction processes. The conventional signal extraction method involves the recording of the so-called stepping curve, where one of the gratings is precisely moved in multiple steps over one period (11). A comparison of two stepping curves acquired with and without a sample in the beam path allows us to extract the attenuating, scattering, and phase-shifting properties of the measured object (11, 13). In a rotating gantry, the controlled movement of gratings is not feasible because instabilities, like centrifugal forces and system vibrations, perturb the system. Therefore, we use the presented alternative signal extraction approach, which involves the modeling of all grating movements in the measured data.

### Interferometer Simulations

In order to find the optimal setup geometry and grating parameters, we use a wave optical simulation based on Fresnel propagation similar to the previous work in refs. 47 and 48. The simulation allows us to estimate the intensity, visibility, and number of fringes on the detector for a given set of system parameters. In contrast to the other mentioned wave optical simulations, here only a one-dimensional wave front is propagated onto a detector row. This still describes the interferometer response to the various parameters sufficiently well and is more efficient in development and execution.

The different gratings, which interact with the wave front during propagation, are implemented closely following their fabrication specifications and their mechanical properties. All relevant geometrical design parameters, such as the period, height, duty cycle, bridge fraction, and grating profile (i.e., the inclination angle and the bending radius), are configurable. For realistic material interactions, the material properties of the substrate, grating lamellae, and polymer-matrix materials are obtained from the xraylib library (49).

Since in the implemented geometry, the gratings are bent cylindrically to focus into the X-ray source spot, the divergence of the radiation can be ignored, and a plane wave model is sufficient. This simplifies the simulation but comes with the drawback that nonperpendicular propagation through the grating structures is not included.

To include the spectral dependencies of the system, the X-ray tube spectrum, the spectral filtration, and the detector efficiency are implemented into the framework. This is done by propagating multiple monochromatic wave fronts in parallel—one for each energy bin—and in the end, integrating the resulting detector signals.

To finally extract the interferometer performance, the simulated G_2_ is stepped over its full grating period, which yields a stepping curve with equidistant sampling points. This curve is then processed as described in ref. 13 and gives us the intensity, visibility, and differential phase of the analyzed parameter configuration. From the differential phase of neighboring pixels, the number of fringes on the detector can be calculated.

Since the extended X-ray source size has an huge impact on the system performance, it is included into the simulation. This is done by considering all G_0_ slits, which contribute to a sampling point in the G_2_ plane. This is usually not required for simulations of laboratory microfocus X-ray tubes because here, only a few slits contribute, which can be simplified to a single slit as an approximation.

By considering this variety of parameters and effects, the simulation achieves realistic results and closely matches the experimental performance.

### Grating Fabrication and Parameters

All gratings were fabricated by the “direct” deep X-ray LIGA process, which involves X-ray lithography with synchrotron radiation and subsequent electroplating with gold (Karlsruhe Institute of Technology and Microworks GmbH) (14, 16, 34). To avoid deformations of the long and fine grating structures, a “bridge” design is used, where the photoresist lamellae are stabilized and interconnected by small photoresist bridges (e.g., Fig. 2 *A* and *B*). The gratings are based on graphite or polyimide substrates, which are, unlike silicon, also suitable for the required small bending radii. G_1_ was fabricated by a slanted exposure of a standard rectangular mask (32) at an angle of $6.7^{{}^{\circ}}$. Together with a height of approximately 18.5  μm, the inclined lamellae yield an effectively triangular height profile if projected perpendicular to the substrate. The grating periods are 4.805, 4.34, and 45  μm for G_0_, G_1_, and G_2_, respectively. In this presented implementation, the grating fabrication technology for the G_0_ limits the system sensitivity because finer periods than 4.8  μm are currently not available in the required quality for hard X-ray spectra. The duty cycles (ratio of gold lamellae width to period) are ∼0.5, and the gold structure heights are around 280  μm for G_0_ and approximately 300 μm for the G_2_ tiles.

### Grating Characterization

The AXT analysis method used for grating characterization provides spatially resolved mapping of several grating parameters and was originally proposed for rectangular, high–aspect ratio absorption gratings (36). However, it is also applicable to our phase grating G_1_ considering two aspects. First, the triangular G_1_ height profile is obtained by the projection of inclined lamellae and not by triangular-shaped grating structures. Second, the limitation of the method to high–aspect ratio absorption gratings was avoided by using a low energy spectrum of 30 kVp. For a structure height of approximately 18.5 μm, this yields sufficient contrast in the AXT measurements. The shape factor is calculated from the period and the duty cycle as well as the lamellae height and inclination relative to the substrate. It hence represents a suitable quality measure, indicating a degree of lamellae inclination. Perfectly rectangular lamellae are described by a value of zero. If the lamellae are tilted such that the projection perpendicular to the substrate yields a triangular height profile, the shape factor is one.

### Shadowing Suppression by Bent Gratings

The absorption X-ray gratings G_0_ and G_2_ produced for this setup via the LIGA process consist of fine lamellae oriented perpendicular to the substrate surface (14). On flat substrates, the high–aspect ratio gratings hence only work as intended in a parallel-beam geometry. In divergent-beam geometry, the incident angle of the rays varies, and due to the height of the absorbing structures, partial absorption leads to intensity and visibility loss. Only X-rays crossing the grating perpendicular to the substrate surface are not affected by shadowing. Consequently, a solution to suppress the shadowing artifact in fan beam geometry is to bend the grating, such that the focal spot of the lamellae coincides with the X-ray source spot (33). In *SI Appendix*, Fig. S3, a rendering visualizes the high aspect ratio and the bending process of the G_0_. With this approach, the usable field of view can be extended while still using high–aspect ratio gratings (50). To achieve small bending radii, flexible substrate materials, such as graphite and polyimide, replaced fragile silicon and glass substrates. For the used one-dimensional grating structures, cylindrical bending of the gratings is sufficient to reduce the shadowing artifact.

### Extended Reference Processing

Fundamentally, the idea of the introduced reference processing is to extract all scan-to-scan persistent information from a sample free scan. In the presented system, the interferometer performance and characteristic vibration patterns are stable over time and thus, are also present in sample scans. For example, the intensity fluctuations follow a certain frequency, amplitude, and pattern. Since the same pattern also appears with the same frequency and amplitude in a following sample scan, its contribution can be corrected during sample processing and reconstruction. A correction solely based on the sample scan would not be possible because differentiation between intensity fluctuation and sample data would be difficult and might lead to cross-talk. During reference processing, the challenge lies in efficiently identifying and separating the occurring patterns in a way that is correctable during sample processing.

As shown in Fig. 3*A*, the reference processing is a multistep approach that optimizes the measured data to the Talbot–Lau interferometry data model. It starts with a standard signal extraction (i.e., phase retrieval), which yields the interferometer intensity *I*_0_, visibility *V*_0_, and phase *P*_0_. While in this early step, the results are greatly corrupted by the vibrations, this result is an initial guess for following steps.

The mechanical oscillations of the system move and deform the gratings relative to each other and hence, induce periodic phase, intensity, and visibility fluctuations. We found that we can efficiently model these changes by low-order two-dimensional polynomial functions and extended the data model accordingly. Since the G_2_ consists of multiple tiles, these have to be modeled individually with tilewise polynomial patterns in this step.

With this extension, visibility and phase fluctuations are already sufficiently well described by the model, and only intensity fluctuations are remaining in the residuum. Using principal component analysis (PCA) on the residuum, the most dominant intensity variation patterns can be further extracted. The resulting intensity correction patterns are depicted in Fig. 3*B* as $R_{i,1-3}$, and the corresponding intensity coefficient per projection $c_{i,1-3}$ is in Fig. 3*D*. Other than the two shadowing-induced intensity patterns $R_{i,2}$ and $R_{i,3}$, there is an all-pixel constant pattern $R_{i,1}$, which corrects global intensity variation (e.g., flux fluctuation of the X-ray tube).

In the final processing step, the data model and the identified correction patterns are compressed using PCA. This step is required to simplify the patterns and reduce the number of free parameters (i.e., polynomial coefficients) during sample processing. Particularly, the tilewise defined polynomial patterns can otherwise not be optimized to a sample scan due to the high risk of fitting the locally defined patterns to the sample. To extract the most dominant visibility and phase variation patterns, all polynomial patterns are summed up and analyzed with PCA. The results are shown in Fig. 3*B*, where again, all-pixel constant patterns $R_{v,1}$ and $R_{p,1}$ are most dominant. For the visibility, only two more patterns are relevant. Similar to the intensity patterns, they mainly represent large gradients, implying that the visibility variations are primarily caused by slight changes in the beam shadowing in the G_0_ grating.

In the resulting phase variation patterns $R_{p,1-6}$, the tilewise nature of the G_2_ is well visible, but as all tiles seem to oscillate in a coupled fashion, the proposed PCA-based compression approach works.

After all these reference processing steps, the interferometer characteristics *I*_0_, *V*_0_, and *P*_0_ are also extracted free of any artifacts and can be used for correction during the sample processing.

While in Fig. 3*B*, the pixelwise scan-to-scan persistent system properties are shown, the per-shot contribution of the individual correction patterns is plotted in Fig. 3*D*. From these coefficients, some scan-to-scan persistent information can also be extracted, in particular the low-frequency behavior, which is, for example, highlighted in Fig. 3*E* in orange, and the frequencies and amplitude of the oscillations. The phase of the coefficients oscillations obviously is unique for each scan and must be optimized during sample processing.

### Sliding Window Signal Extraction

The sliding window signal extraction is a compromise between feasibility and performance for systems that continuously rotate (19). A window of several consecutive projections is converted to a single intensity and visibility projection located at the center of the processed set of projections. To minimize motion artifacts (i.e., visible in Fig. 3*J* as low-frequency noise-like structures) due to the effect that the projection angle changes in subsequent projections, the window size needs to be small. On the other hand, the window should be sufficiently large to ensure good phase sampling over ideally 2π. At this point, our setup leverages the high-frequency phase variation from Fig. 2*H*. The oscillation guarantees adequate phase sampling over at least 1.5π within a sufficiently small window size of only 10 projections. If the intrinsic vibrations of the setup would not be in the required range, either an actuator or further stabilization of the setup would be required. Since this approach combines multiple projections to one, the resolution of the tomographic reconstruction is reduced because particularly sharp edges are difficult to recover in this process without any artifacts. However, this drawback is accepted as the loss in resolution is insignificant and spatial resolution is currently not the primary objective. From a computational perspective, the method is advantageous because it is an extremely fast signal extraction method as it is a linear problem, which can be solved analytically.

In dark-field imaging, the energy dependence of the interferometer performance leads to an artifact similar to beam hardening in conventional attenuation contrast (51). By measuring a nonscattering material like POM or water, a beam-hardening correction is generated. This correction is applied prior to the FBP reconstruction (37) of all presented images. As an example of the signal extraction results prior to reconstruction, the transmission signal and dark-field signal sinograms of the cylinder phantom (Fig. 4 *A*, *D*, and *G*) are shown in *SI Appendix*, Fig. S5.

### Dark-Field Signal Calibration

The dark-field signal strength depends on the feature size of the small-angle scattering structures as well as on several system-specific parameters, like the interferometer sensitivity or the X-ray spectrum (9). Essentially, the reconstructed values can be calibrated to what we refer to as HUd. Similar to the attenuation Hounsfield scale, this should ensure a quantitative consistency between different dark-field CT systems (4). We select neoprene foam and air as reference calibration materials. The neoprene is a suitable material to model the microstructure of lung alveoli (*SI Appendix*, Fig. S6) (38), featuring a very weak attenuation but strong dark-field signal. However, further evaluation on long-term stability is required by future work. The HUd scale is defined by a value of 1,000 for neoprene as a strongly scattering material and a value of 0 for air and every other nonscattering material. This gives a similar image impression as attenuation images since regions with lower scattering density are darker. Especially for imaging of the lung, the use of positive numbers for HUd ensures a clear differentiation to the attenuation Hounsfield units, which are mostly negative for lung tissue. In contrast to radiography dark-field systems, the presented CT implementation and subsequent signal calibration enable quantitative imaging. While radiography systems come with image obstruction from the ribs and varying anatomical shape of the chest or lung, which can make evaluation difficult, this problem is solved by our system. The tomographic reconstruction and proposed calibration make sure that the signal strength is reproducible and free of overlapping structures.

### CT System and Dose Estimation

The dark-field CT prototype is based on a state-of-the-art Philips Brilliance iCT SP system. The gantry is equipped by default with an air bearing, which ensures a low level of vibrations on the rotating gantry (29). The presented scans were acquired in axial mode using a spectrum of 80 kVp and a tube current of 550 mA. With an unmodified CT system, these settings would lead to a volume computed tomography dose index (CTDI${}_{\text{vol}}$) of 13 mGy. Considering that G_0_ absorbs about 50% of the generated X-ray flux before the radiation reaches the patient, we estimate that the CTDI${}_{\text{vol}}$ is around 7 mGy for the demonstrated results from the dark-field CT prototype. This dose value lies within the clinically applicable range for chest CT of adults with state-of-the-art CT systems (52).

For the reconstructions, we used all 2,400 projections acquired during a single gantry rotation with 1 s rotation time. The presented implementation uses 32 detector lines and covers more than 90% of the original detector columns. This results in a reconstruction volume that is 450 mm in diameter. Extending the setup to 64 and more detector lines is possible by upgrading to G_2_ gratings with more coverage.

Since the G_0_ and G_1_ gratings were integrated into the collimator box, the original bowtie filter had to be removed. While the partial shadowing of the large X-ray focal spot in the G_0_ results in a similar intensity modulation, future dark-field CT can be optimized by adding a specifically designed bowtie filter for optimal dose reduction and artifact suppression.

## Supplementary Material

Supplementary File

## Data Availability

All study data are included in the article and/or *SI Appendix*.
