# Supplementary material for: Dark-field computed tomography reaches the human scale
Source: Proc Natl Acad Sci U S A. 2022 Feb 7;119(8):e2118799119. doi: 10.1073/pnas.2118799119 (PMC8872773; doi:10.1073/pnas.2118799119)
Supplement: Supplementary File [file pnas.2118799119.sapp.pdf]

PNAS
www.pnas.org

Supplementary Information for

Dark-field computed tomography reaches the human scale

Manuel Viermetz, Nikolai Gustschin, Clemens Schmid, Jakob Haeusele, Maximilian von Teuffenbach, Pascal Meyer, Frank Bergner, Tobias Lasser, Roland Proksa, Thomas Koehler, and Franz Pfeiffer

Manuel Viermetz.
E-mail: manuel.viermetz@tum.de

This PDF file includes:

Figs. S1 to S7 (not allowed for Brief Reports)

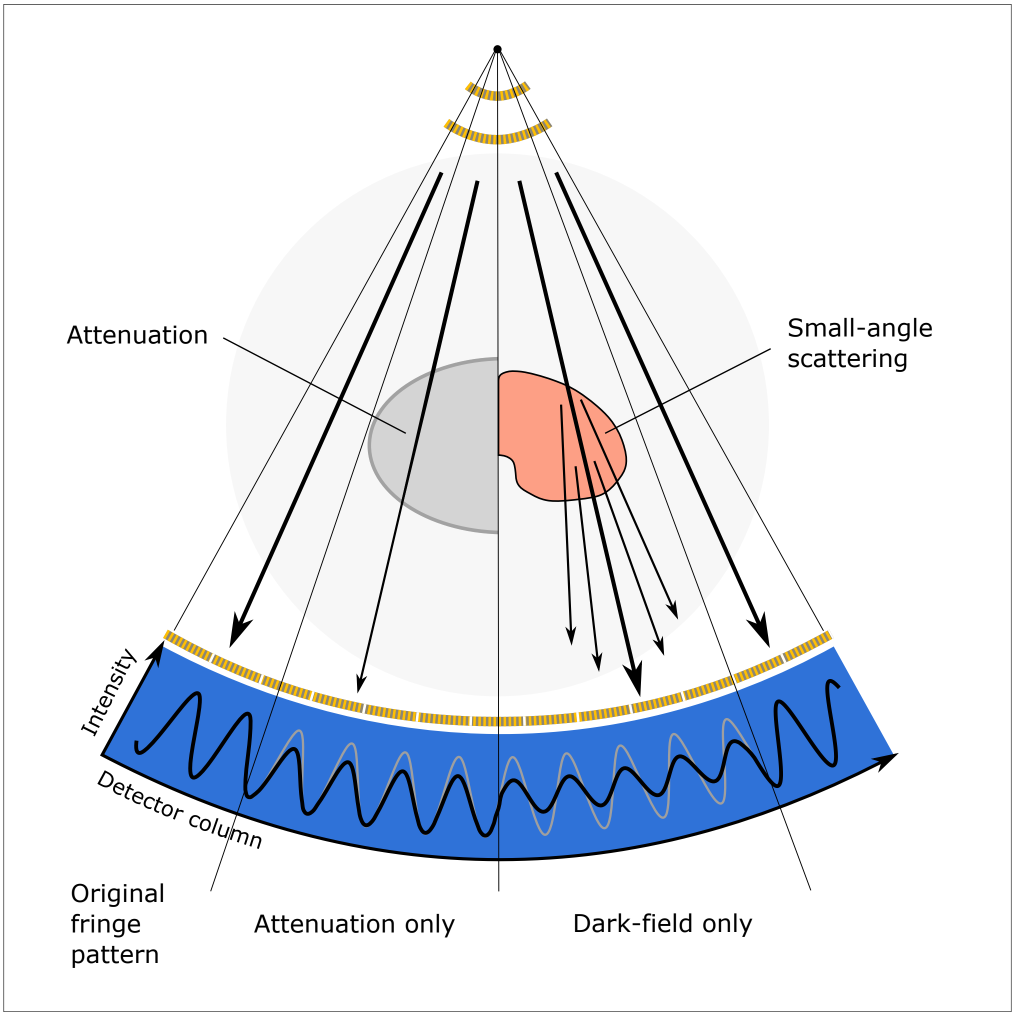

Fig. S1. Contrast signals in a Talbot-Lau interferometer. In absence of any sample the fringe pattern remains unchanged. In case of a purely attenuating sample (left) the attenuation signal is a reduction of the mean fringe intensity. A purely scattering sample (right) causes a visibility reduction of the fringe pattern while the mean value stays constant. This is referred to as the dark-field signal. The figure is simplified and does not include the different interaction path-lengths.

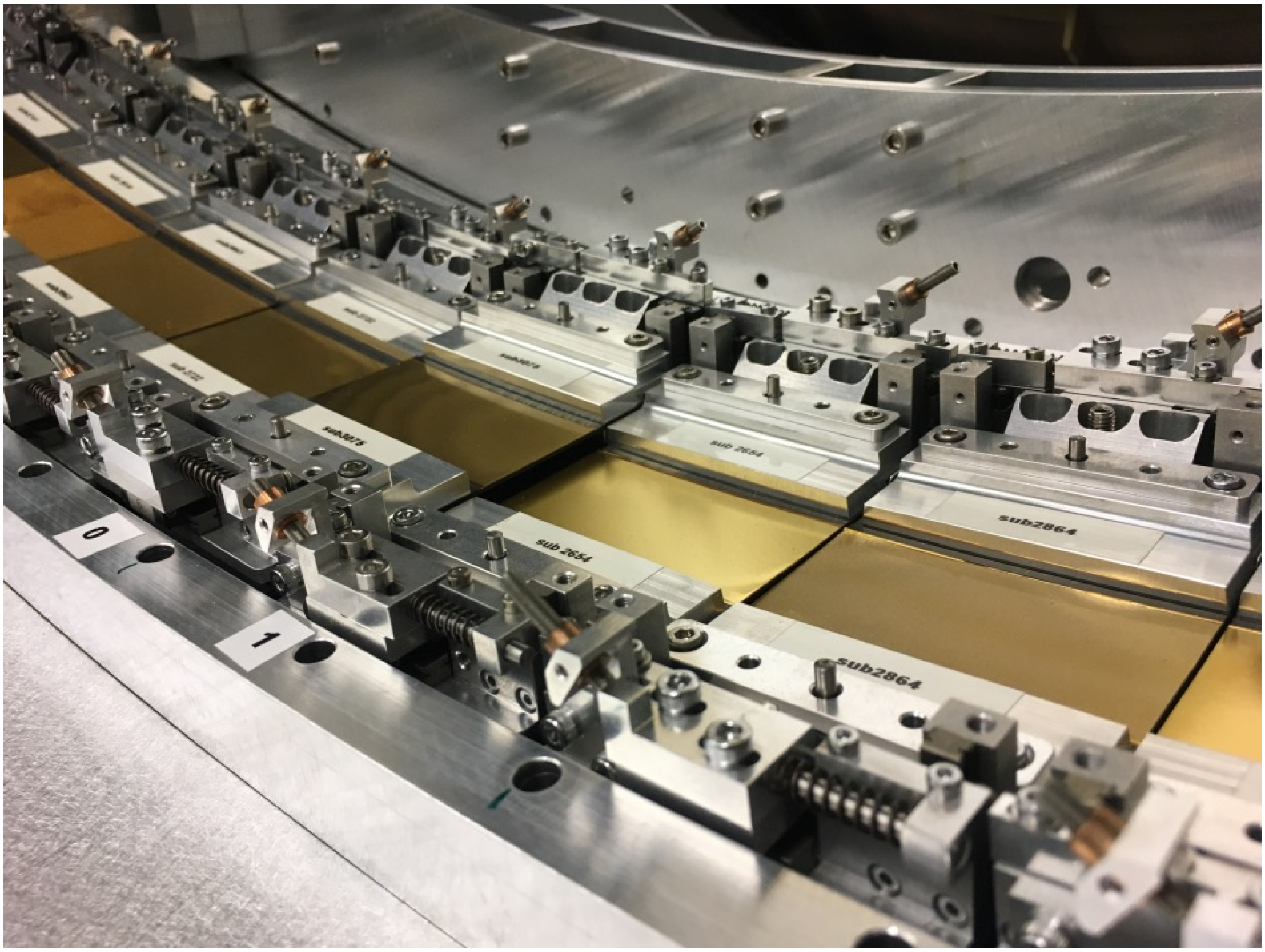

Fig. S2. Mounting of the G_2 grating tiles on the detector arc. The entire G_2 consists of 13 tiles. Due to the fan-beam geometry of the setup each individual tile must be bent to the appropriate radius as visible in this close-up image. During installation the G_2 tiles are aligned in the interferometer by adjusting their position in beam direction and rotation around the beam axis (precision better than 100 µm and $0.05°$, respectively). Furthermore, the gap between adjacent tiles must be minimized (gap size < 100 µm) to reduce stitching artefacts. Specialized mounts allow the positioning and rotation in the required directions as well as a flexible replacement of individual tiles. As visible in the picture tile 0 is currently elevated above the surrounding tiles and large gaps between the tiles are apparent.

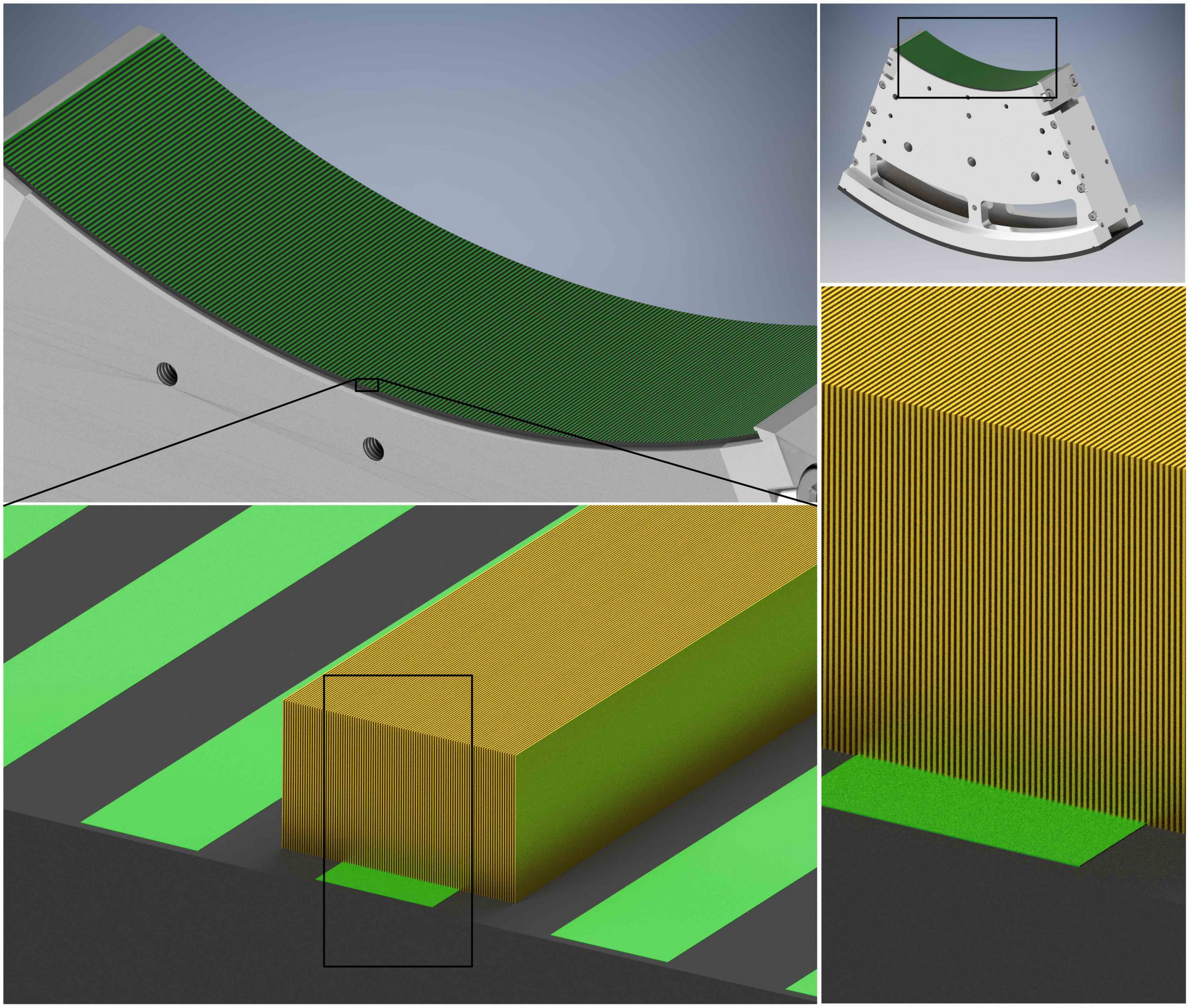

Fig. S3. Rendering of the bent G_0 grating installed on the mount. Several renderings are shown to visualize the required aspect ratio of the G_0 grating structures. In green dummy grating lines illustrate the general layout and orientation of the grating lamellae but not the correct periodicity. In the two lower detail enlargements 100 grating lamellae in realistic periodicity and aspect ratio are depicted. Comparable to the G_0 shown in Figure 2a, the height of the lamellae is 280 µm with a periodicity of 4.8 µm. To keep the renderings simple, we omitted details such as the polymer matrix, in which the lamellae are embedded, and the polymer bridges, which are required for stabilization during the fabrication process.

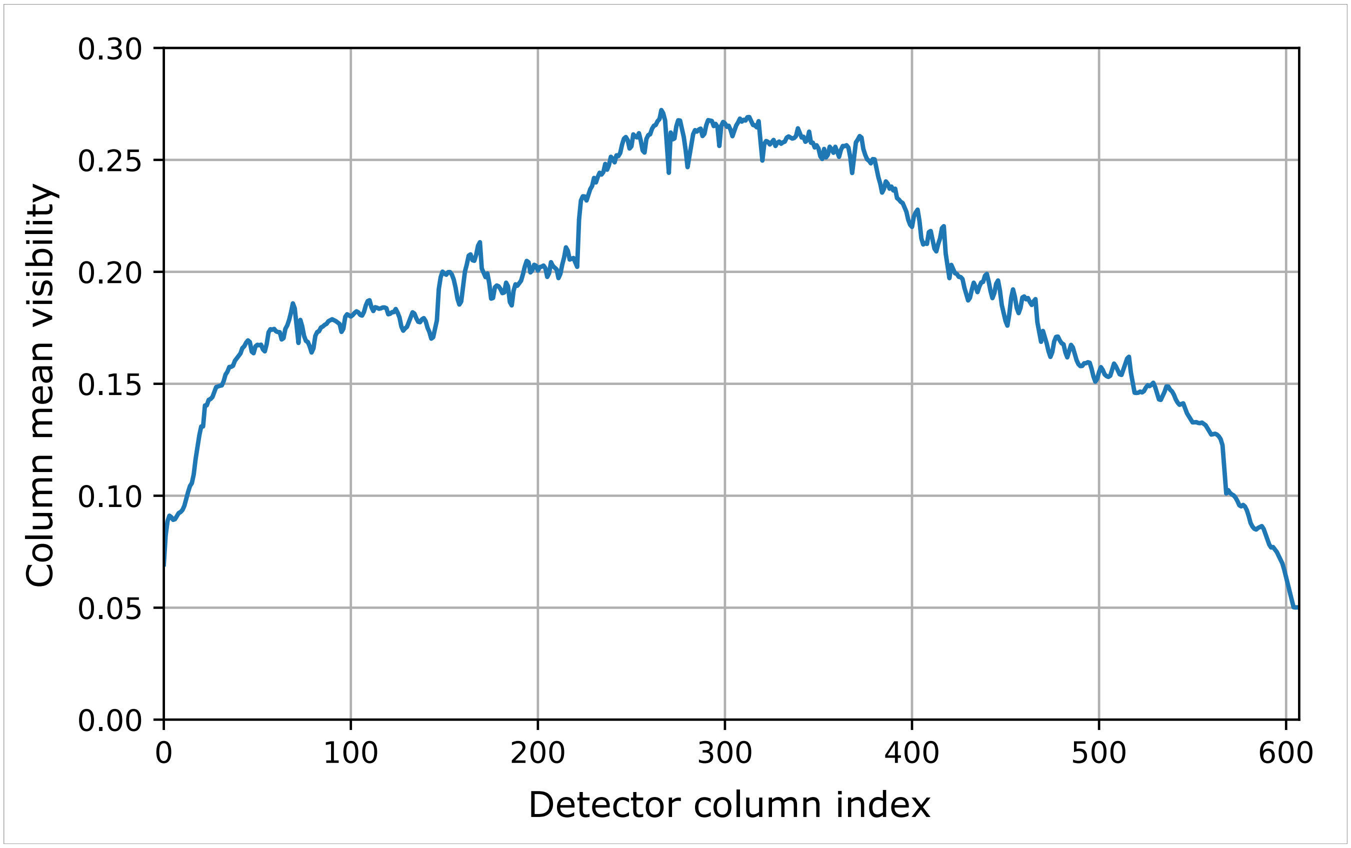

Fig. S4. Column mean visibility for system characterization. Derived from the colour coded visibility in Figure 2f this plotted version shows the centered wide performance maximum of the implemented Talbot-Lau interferometer. The central region performs well with a mean visibility of around 25% which is most important for tomographic reconstruction since here most sample information is measured. Towards the outer regions the performance reduces due to increasing projected X-ray source width. This leads to partial shadowing in the G_0 grating and consequently to intensity and visibility loss.

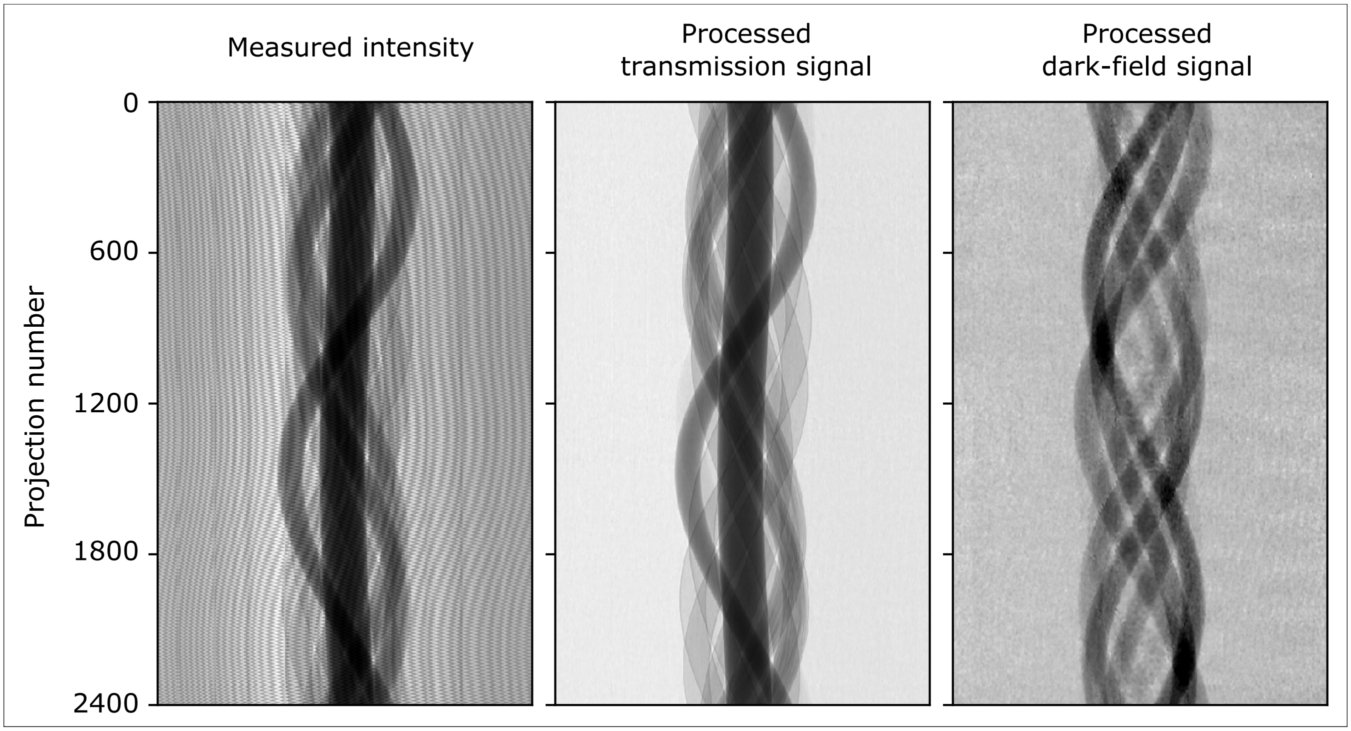

Fig. S5. Sinograms of the raw data and the corresponding processed signals. 2400 projections are acquired during one full 360° rotation in one second. Vibrations on the CT gantry cause the fringe pattern to move during the acquisition which can be observed as the fine sinusoidal-like structure in the plotted intensity sinogram. With the proposed sample processing pipeline the transmission and the dark-field signal can be extracted from the measured projections resulting in the two sinograms shown on the right. At this point it is already possible to differentiate some materials. For instance, the central highly attenuating object, which is clearly apparent in the transmission as a vertical line, gives no dark-field signal.

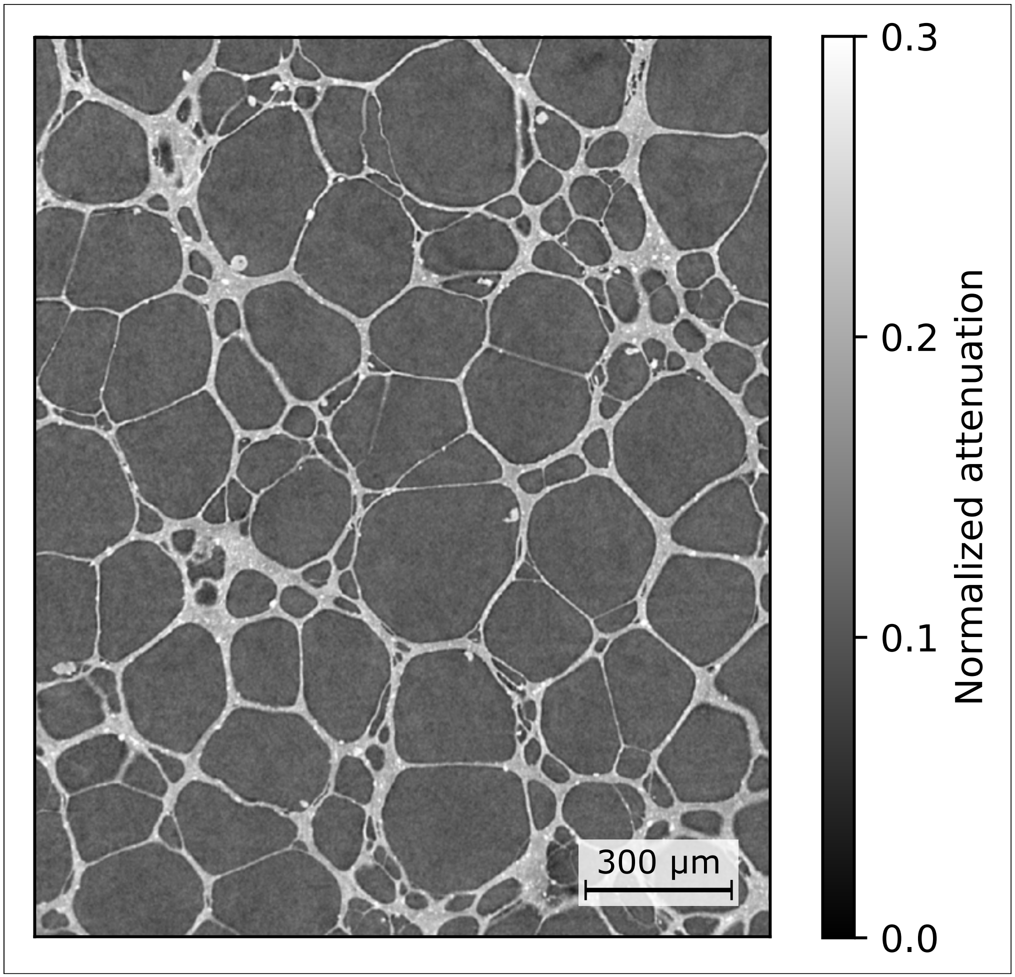

Fig. S6. MicroCT slice of the closed-cell neoprene phantom material. With a microstructure similar to pulmonary alveoli, the material is a suitable phantom to mimic the attenuation and scattering properties of the human lung. The image was acquired with a ZEISS Xradia Versa XRM-500 (Zeiss, Oberkochen, Germany) microCT system and features a pixel size of 1.62 µm.

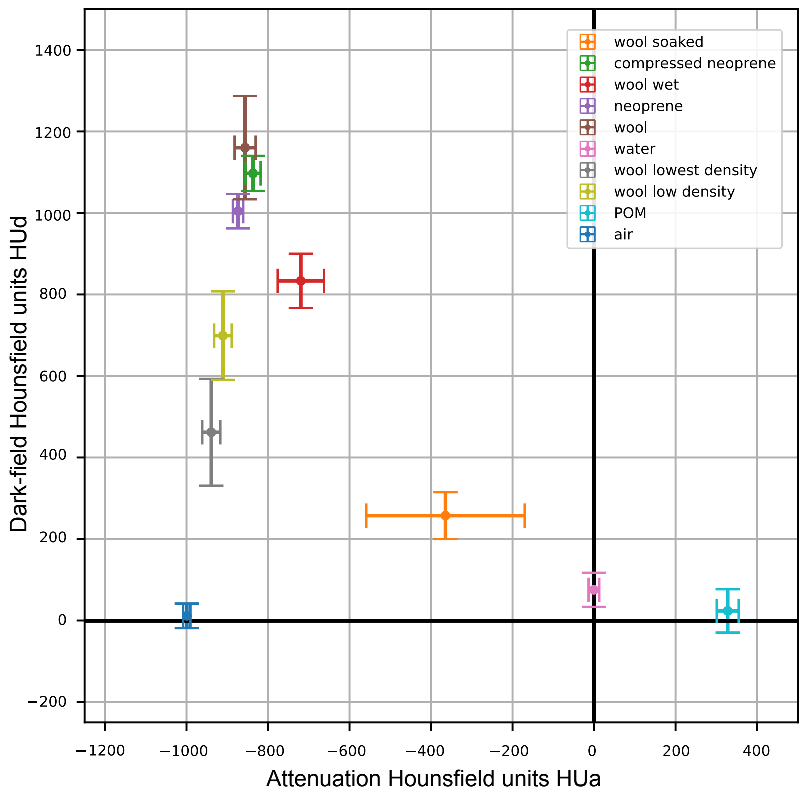

Material	HUa		HUd	
Water	0±9	→	62±84	↘
Air	-1000±6	↘	-2±53	↘
Neoprene	-875±9	↘	1000±73	↗
Neoprene compressed	-838±13	↘	1147±73	↗
POM	327±18	↗	-16±83	↘
Powdered sugar	-244±79	→	1845±114	↗
Wool (dry)	-854±21	↘	1122±258	↗
Wool moist with water	-715±48	↘	862±117	→
Wool soaked with water	-370±164	→	252±110	↘
Wool low density	-912±20	↘	725±155	→
Wool lowest density	-940±18	↘	478±198	↘
Bone (synth.)	432±67	↗	1618±277	↗

Fig. S7. Quantitative material parameters retrieved from the cylinder phantom reconstruction. Scatter plot of the material properties extracted from the various materials of the cylinder phantom shown in Figure 4a, d and g. Standard deviations in the HUd unit are larger than for the corresponding HUa values. This is related on the one hand to the generally lower signal to noise ratio in the dark-field signal, and on the other hand to the inhomogeneity of the analysed materials. Therefore, the signal deviates significantly. The arrows in the table indicate a qualitative classification of the signal within the overall measured signal range of the respective contrast modality.
